# Effects of artichoke (*Cynara scolymus*) leaf extract on the growth, blood, and biochemistry parameters of Nile tilapia (*Oreochromis niloticus*)

**DOI:** 10.1007/s11250-025-04536-y

**Published:** 2025-06-25

**Authors:** Ramazan Esen, Mustafa Öz, Suat Dikel

**Affiliations:** 1 Department of Fisheries and Diseases, Graduate School of Health Sciences, Aksaray University, Aksaray, Türkiye; 2 Department of Fisheries and Diseases, Faculty of Veterinary Medicine, Aksaray University, Aksaray, Türkiye; 3 Department of Aquaculture, Faculty of Fisheries, Cukurova University, Adana, Türkiye

**Keywords:** Nile Tilapia (*Oreochromis niloticus*), Artichoke Leaf Extract (*Cynara scolymus*), Growth performance, Sustainable Aquaculture, Feed additive

## Abstract

In this study, artichoke leaf extract (ALE) was added to the Nile tilapia diet at different ratios (0.00%, 1.00%, 2.00%, and 3.00%) for 30 days to investigate its effects on growth performance, hematological parameters, and blood biochemistry. A total of 240 fish with an average initial weight of 33.9 ± 1.14 g were used. The results showed that ALE supplementation significantly improved growth performance, with the best results observed at 2.00% inclusion (final weight: 58.01 ± 0.18 g, FCR: 1.48 ± 0.02, SGR: 1.79 ± 0.01) (*p <* 0.05). Hematological analysis revealed an increase in hemoglobin (Hb) levels (control: 9.18 ± 0.62 g/dL; 2.00% ALE: 12.83 ± 1.19 g/dL) and a decrease in white blood cell count (control: 3.54 ± 0.06 m/mm^3^; 2.00% ALE: 3.39 ± 0.07 m/mm^3^) (*p <* 0.05). Blood biochemistry results indicated a significant reduction in glucose (control: 73.67 ± 4.16 mg/dL; 2.00% ALE: 51.00 ± 2.00 mg/dL) and cholesterol levels (control: 227.67 ± 6.11 mg/dL; 2.00% ALE: 117.67 ± 3.21 mg/dL) (*p <* 0.05), suggesting improved metabolic balance. The findings indicate that 2.00% ALE supplementation enhances growth performance and may positively influence fish health by modulating blood parameters, making it a promising natural additive for sustainable aquaculture.

## Introduction

The increasing global population and heightened consumer awareness have led to a growing demand for aquatic products. Since capture fisheries face limitations due to finite natural resources, aquaculture has emerged as the primary alternative, experiencing rapid expansion. To ensure its sustainability, it is crucial to develop innovative aquaculture models that reduce water pollution and incorporate environmentally friendly feed alternatives.

Nile tilapia (*Oreochromis niloticus*) occupies an important position in the aquaculture sector and is emerging as a vital contributor to global fish production. With its adaptability, rapid growth, and versatile culinary characteristics, this freshwater fish species is recognised as one of the most economically important and widely farmed species worldwide (Bonham 2022). Nile tilapia farming is increasingly affected by disease outbreaks, rising feed expenses, and ecological issues stemming from nutrient overloading in culture environments (El-Sayed and Fitzsimmons 2023). Synthetic feed additives and antibiotics have traditionally been used to enhance growth and disease resistance, but their long-term sustainability remains questionable (Tacon et al. 2022). Therefore, exploring natural plant-based additives, such as ALE, may provide an environmentally friendly alternative to improve tilapia health and production efficiency.

Artichoke (*Cynara scolymus*), a plant widely cultivated in Mediterranean regions, is recognized for its high antioxidant content and various pharmacological properties. Recent studies on animal models have demonstrated that artichoke leaf extract (ALE) exhibits hepatoprotective, antioxidant, and anti-inflammatory effects, making it a potential candidate for improving health and metabolic function (Azzani et al. 2007).

While ALE’s hepatoprotective, antioxidant, and antimicrobial effects have been well-documented in mammals (Shallan et al. 2020; Biel et al. 2020), recent studies have also begun to explore its potential applications in aquaculture species. Kia et al. (2025) reported that dietary ALE enhanced immune parameters and antioxidant enzyme activities in common carp (*Cyprinus carpio*), suggesting its role as a natural immunostimulant. Similarly, Shohreh et al. (2025) demonstrated that ALE supplementation in Nile tilapia diets significantly improved growth, digestive enzyme activities, hepatic antioxidant status, and stress resistance under heat challenge. Elsayyad et al. (2024) also suggested ALE's neuroprotective and antioxidative role in Nile tilapia under fluoride exposure, though research on its dietary inclusion and long-term effects in aquaculture systems remains limited.

Artichoke extract has been investigated for its potential benefits in fish. Studies have shown that the penetration of pomegranate peel and artichoke leaf extracts into fish tissue causes browning of fish, indicating a potential effect on the quality of marinated fish fillets (Essid et al. 2020). These findings reveal that artichoke extract may benefit fish, particularly by contributing to antioxidant capacity and improving tissue quality. Several plant-based feed additives, including garlic oil, pomegranate peel extract, and black cumin oil, have been investigated for their effects on fish growth and immunity (Badrey et al. 2019; Öz et al. 2024a, b). These additives enhance metabolic function, improve gut health, and support antioxidant enzyme activity (Ngugi et al. 2017; Pastorino et al. 2022). Given its high polyphenol content and known hepatoprotective properties, ALE may offer similar or even superior benefits as a natural growth enhancer and immunostimulant in Nile tilapia diets.

Artichoke cultivation areas have been increasing in recent years, and approximately 60% of artichoke is discarded as residues (outer leaves, leaves, and stems) after processing in the industry. These residues are used as fertilizer or animal feed (Savlak et al. 2020).

This study aimed to evaluate the effects of dietary supplementation with different concentrations of artichoke (*Cynara scolymus*) leaf extract (ALE) on the growth performance, hematological indices, and selected biochemical markers of Nile tilapia (*Oreochromis niloticus*), with a focus on its potential as a natural, sustainable feed additive.

## Material and method

### Fish material and experimental design

This study was conducted within the framework of the ethical approval obtained from Local Ethics Committe on 03.06.2022 and in accordance with the principles of the Local Ethics Committee. In the study, 240 fish with an average initial body weight of 33.9 ± 1.14 g were used. The research was carried out in 80-L aquariums with 20 fish each in 3 replicates, and 60 fish were used for each group. This research was carried out with 240 fish in total. In order to maintain a consistent water temperature across all experimental groups during the duration of the study, an Eheim brand 100-Watt thermostat heater was utilised, ensuring that the water temperature remained stable. During the experimental period, the oxygen content and water temperature of the aquarium water were checked twice daily using an OxyGuard oxygen meter (OxyGuard, Birkerød, Denmark). The water temperature was 25 °C and the oxygen content was 4.9–5.2 mg/L during the experimental period. Additionally, the air diffusers and internal filters in the aquariums were cleaned frequently. Throughout the duration of the investigation, the fish in the experiment were nourished with tilapia feed manufactured by a commercial brand. The feed content is given in Table 1. The ALE used in the experiment was obtained from a commercial company (Immunat; TR-48-K-019618). ALE was added to fish feeds by spraying at the rates of 0.00%, 1.00%, 2.00%, and 3.00%. The selected doses were determined based on the literature (Erbaş 2013) and chosen to evaluate a potential dose-dependent effect. A stepwise increase was applied to observe whether the extract exhibits a linear or nonlinear biological response, while avoiding potential toxicity or inefficacy at extreme doses. Fish food was prepared in 100 g batches and stored in the refrigerator at 4 °C. The active ingredient was sprayed into the feeds and then coated with 2 ml sunflower oil. The addition of 2 ml of oil was also made to the control group feeds so that the fat ratios of the research feeds were not different.

**Table 1:** Nutritional value of tilapia feed used in the experiment

Feed Content	
Diet Composition	Average
Crude protein (%)	39
Lipid (%)	6.7
Crude cellulose (%)	4.30
Ash (%)	6.79

## Feeding protocol and method

The fish brought to the research unit were weighed at the end of the 15-day adaptation period, and the research was started by taking the averages. The fish were fed two meals daily at 08:30 in the morning and 17:00 in the evening for 30 days. Water temperatures were checked in the morning and evening with digital thermometers placed in the aquariums. The air stones were cleaned at least twice a week for regular distribution of the air coming from the air motors, and the oxygen content of the water was measured once a day using an OxyGuard brand oxygen-meter after feeding. The average weights of the fish were taken twice during the feeding period, and the experiment was completed at the end of day 30.

## Measurement of fish growth parameters

After the feeding period was completed, the length and weight of the harvested fish were measured meticulously. Using the measurement results obtained as a result of the research, Specific Growth Rate—SGR (Company et al. 1999), Feed conversion ratio—FCR (Santinha et al. 1999), Protein efficiency ratio—PER, (Skalli and Robin 2004), The condition factor -CF (Arellano-Martínez and Ceballos-Vázquez 2001) and Hepatosomatic index-HSI (%) (Cheng et al. 2006) were calculated.

## Blood sampling and analysis

**Fig. 1:**
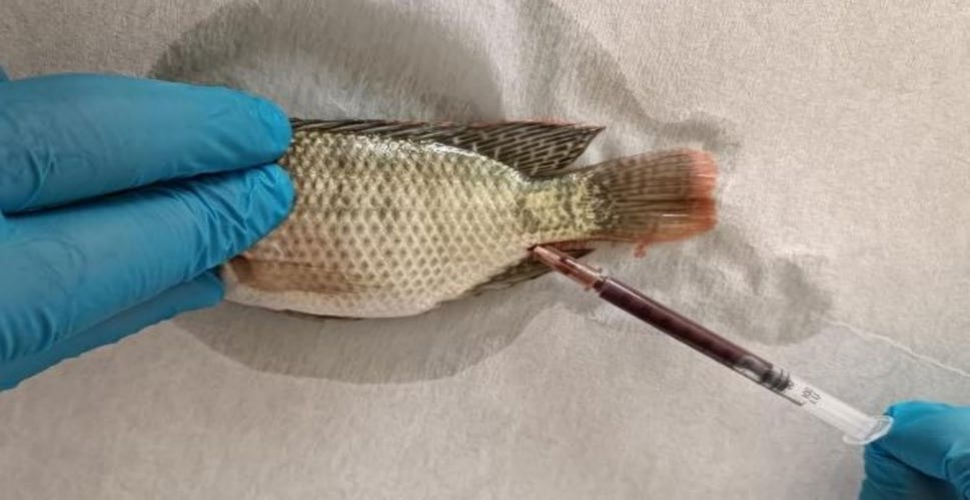
Taking blood from Nile tilapia

Upon completion of the feeding experiment, the fish were subjected to anaesthesia using a concentration of 300 ppm of 2-phenoxyethanol. Subsequently, the fish were promptly cleaned with a solution of 70% ethanol. Following the cleaning process, blood samples were collected from the caudal vein using syringes that were pre-treated with heparin (Fig. 1). For haematological analysis, the blood sample was separated into conventional lavender-top blood collection tubes containing anticoagulant (EDTA). Additionally, standard red-top (SST™ II) advance serum separator tubes were used to analyse serum biochemical characteristics. The samples underwent centrifugation at a speed of 13,000 × g at a temperature of 4 °C for a duration of 10 min in order to get serum. The haematological parameters were assessed promptly, whilst the serums were preserved at a temperature of −80˚C until the biochemical parameters could be analysed. WBCs were counted using a counting chamber. The haematology auto analyzer MS4-S (Melet Schloesing Laboratories, Osny, France) was used to analyse red blood cells (RBCs), mean cell volume (MCV), mean cell haemoglobin (MCH), MCHC, hematocrit (Hct), and haemoglobin (Hb). A manual haematological examination, following the method of Blaxhall and Daisley, was conducted on all blood samples collected in K3EDTA tubes to verify the accuracy of the automated blood count equipment results (Blaxhall and Daisley 1973). Alkaline phosphatase (ALP), Aspartate aminotransferase (AST), Alanine aminotransferase (ALT), Glucose (GLU), Phosphorus (P), Calcium (Ca), Albumin (ALB), Cholesterol (CHOL), Creatine (CRE), Total protein (TP) and Globulin (GLOB) were tested. They were measured by Melet Schloesing MScan II biochemical analyzer (Osny, France).

## Statistical analyses

Statistical analyses were performed using SPSS 18.0 (SPSS Inc., Chicago, IL. USA) to determine the differences between the groups in growth parameters and changes in blood parameters of fish fed with feed supplemented with ALE at different rates.

## Results

At the end of the study, the effects on growth parameters, blood parameters, and blood biochemistry of Nile tilapia (*Oreochromis niloticus*) fed with diets supplemented with ALE at different ratios were investigated.

## Growth parameters and their effects on indices

**Fig. 2:**
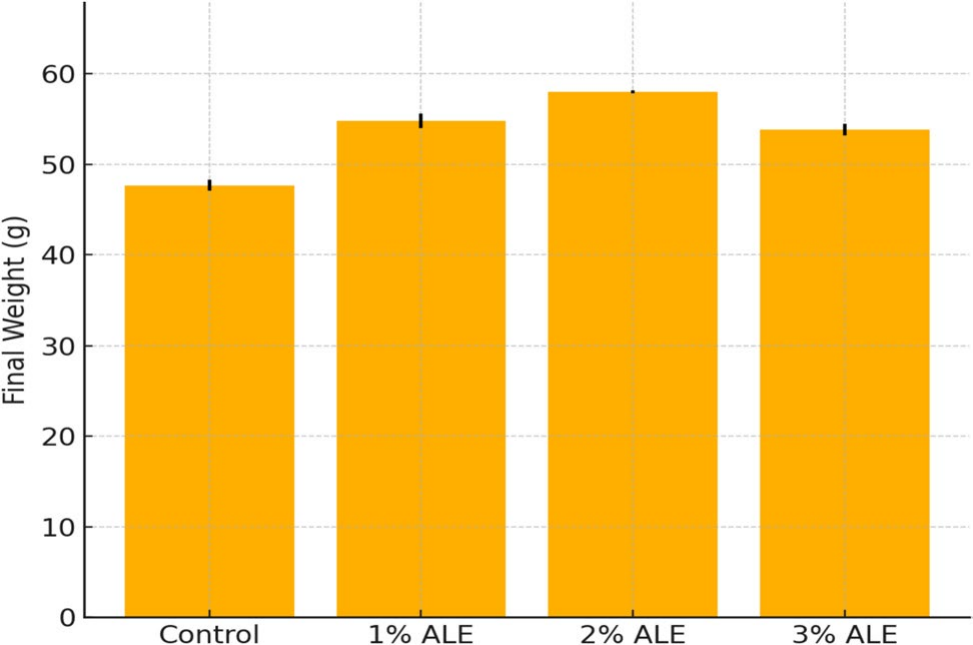
Final body weights of Nile tilapia fed diets supplemented with different levels of artichoke leaf extract (ALE). Values are expressed as mean ± standard deviation (SD). Different letters indicate statistically significant differences among groups (*p <* *0.05*)

In the present study, the effects of ALE added to Nile tilapia (*Oreochromis niloticus*) feed at rates of 0.00%, 1.00%, 2.00%, and 3.00% on the growth parameters of the fish were calculated. To this end, fish with an average initial weight of 33.9 ± 1.14 g were used, and at the end of the 30-day feeding period, the fish reached final weights of 47.69 ± 0.6 g, 54.79 ± 0.8 g, 58.01 ± 0.18 g and 53.84 ± 0.63 g, respectively (Fig. 2 and Table 2.).

**Table 2:** Growth performance of Nile tilapia fed with diets supplemented with different ratios of artichoke leaf extract

	G1-Control	G2-%1 Eng	G3-%2 Eng	G4-%3 Eng
IW	33.9 ± 1.14	33.9 ± 1.14	33.9 ± 1.14	33.9 ± 1.14
FW	47.69 ± 0.6^c^	54.79 ± 0.8^b^	58.01 ± 0.18^a^	53.84 ± 0.63^b^
WG	13.79 ± 0.6^c^	20.89 ± 0.82^b^	24.11 ± 0.18^a^	19.94 ± 0.64^b^
FCR	2.08 ± 0.09^a^	1.66 ± 0.05^b^	1.48 ± 0.02^c^	1.77 ± 0.07^b^
SGR	1.14 ± 0.04^c^	1.60 ± 0.04^b^	1.79 ± 0.01^a^	1.54 ± 0.04^b^
PER	1.23 ± 0.06^d^	1.54 ± 0.05^b^	1.72 ± 0.02^a^	1.45 ± 0.06^c^
FI	28.75 ± 0.23^c^	34.66 ± 0.33^b^	35.85 ± 0.63^a^	35.28 ± 0.33^ab^
DFI	0.95 ± 0.01^c^	1.15 ± 0.01^b^	1.20 ± 0.02^a^	1.18 ± 0.01^ab^
CF	1.57 ± 0.05^b^	1.65 ± 0.06^a^	1.48 ± 0.04^c^	1.58 ± 0.06^b^
HSI	2.38 ± 0.05^a^	2.14 ± 0.07^b^	2.10 ± 0.07^b^	1.99 ± 0.04^c^
VSI	9.70 ± 0.18^a^	9.77 ± 0.48^a^	9.64 ± 0.26^a^	9.73 ± 0.30^a^
GSI	1.08 ± 0.27^b^	1.08 ± 0.21^b^	1.32 ± 0.14^a^	1.32 ± 0.12^a^

G1(Control): 0.00% artichoke leaf extract; G2: 1.00% artichoke leaf extract; G3: 2.00% artichoke leaf extract; G4: 3.00% artichoke leaf extract. The averages expressed using different letters in each row are significantly different (*p <* 0.05). IW: Initial fish weight, FW: Final weight, FI: Feed intake, DFI: Daily feed intake, FCR: Feed conversion rates, SGR: Specific growth rate, PER: Protein efficiency ratio, CF: condition factor, GSI: Gonadosomatic index, VSI: Viscerosomatic index, HSI: Hepatosomatic index

ALE added to the fish feed affected the feed conversion ratio of Nile tilapia and showed a statistical difference between the control and experimental groups (*P <* 0.05). When the feed conversion ratios of the groups were analysed, the best result was observed in the group with 2.00% ALE (G3: 1.48 ± 0.02). On the other hand, the feed conversion ratio was calculated as 2.08 ± 0.09 in the control group, 1.66 ± 0.05 in the group with 1.00% extract (G2) and 1.77 ± 0.07 in the group with 3.00% extract (G4). ALE supplementation significantly improved the feed conversion ratio (*P <* 0.05) compared to the control group. It was determined that ALE added to the diet affected the specific growth rate of Nile tilapia, and there was a statistical difference between the control and experimental groups (*P <* 0.05). When the specific growth rates of the research groups were analysed, the best result was observed in the group with 2.00% ALE (G3: 1.72 ± 0.02) while the specific growth rate was calculated as 1.23 ± 0.06 in the control group, 1.54 ± 0.05 in the group with 1.00% extract (G2) and 1.45 ± 0.06 in the group with 3.00% extract (G4).

**Fig. 3:**
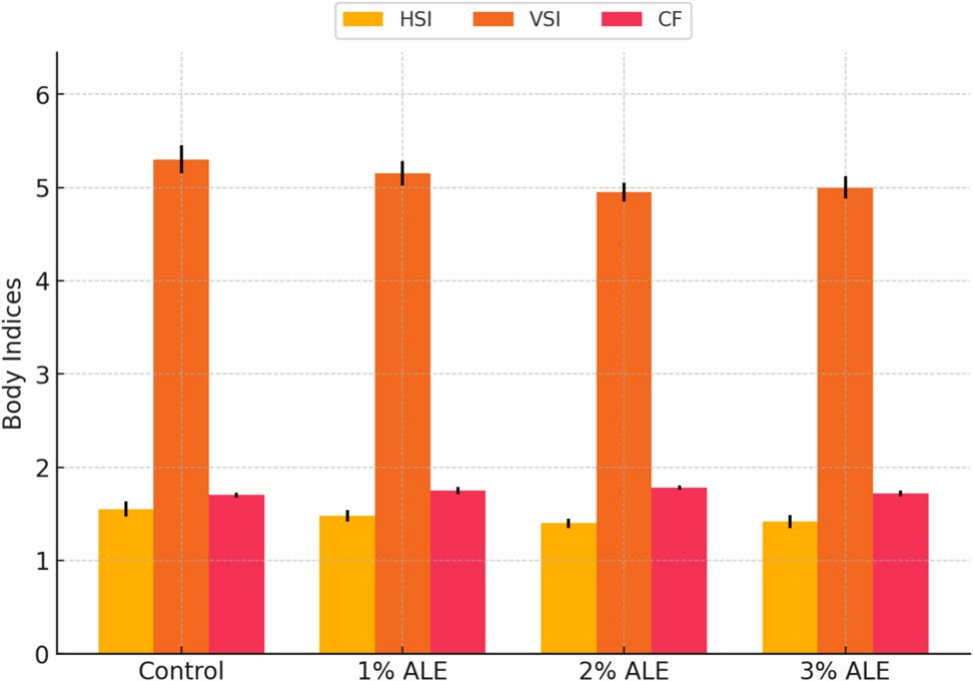
Effect of dietary supplementation with artichoke leaf extract (ALE) on body indices of Nile tilapia. HSI: Hepatosomatic index, VSI: Viscerosomatic index, CF: Condition factor. Data are presented as mean ± standard deviation (SD). Different letters indicate statistically significant differences among groups (*p <* *0.05*)

HSI (hepatosomatic index), Viserosomatic index and Gonadosomatic index values of Nile tilapia were calculated. ALE added to fish feed decreased the HSI value of Nile tilapia and increased the Gonadosomatic index but had no effect on the Viserosomatic index (Fig. 3).

## Effects of ALE on blood parameters

**Fig. 4:**
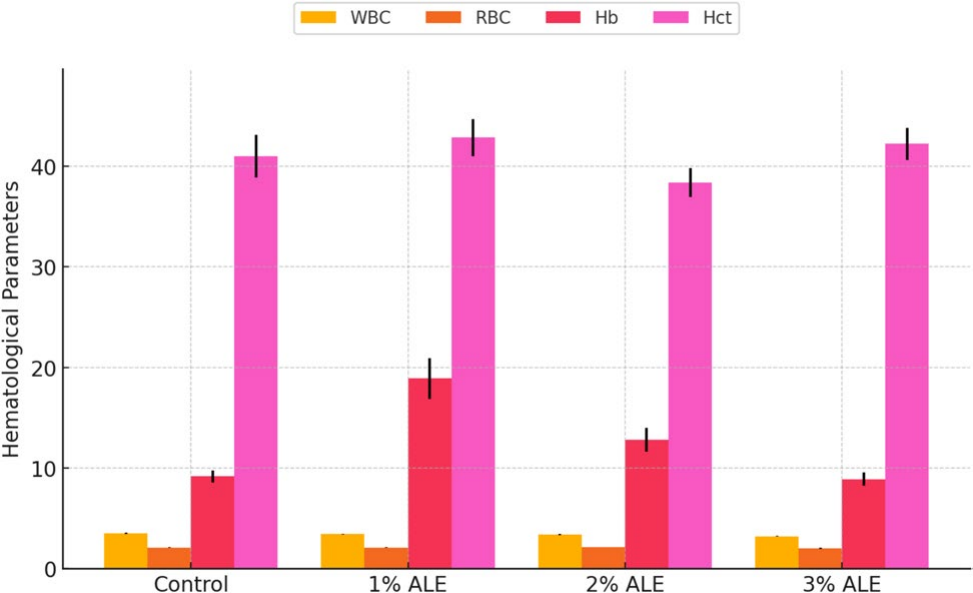
Effect of dietary supplementation with artichoke leaf extract (ALE) on hematological parameters of Nile tilapia. WBC: white blood cell count, RBC: red blood cell count, Hb: hemoglobin, Hct: hematocrit. Data are presented as mean ± standard deviation (SD). Different letters indicate statistically significant differences among groups (*p <* *0.05*)

The evaluation of hematological and serum biochemical parameters provides critical insights into the physiological and metabolic status of fish, aiding in the detection of metabolic disorders and health conditions. In this study, the hematological parameters WBC, RBC, Hb, and Hct were assessed (Fig. 4), while the biochemical parameters glucose, total protein, and albumin were analyzed (Tables 3 and 4).

**Table 3:** Blood parameters of Nile tilapia fed with different ratios of ALE for 30 days

	G1-Control	G2-%1 ALE		G3-%2 ALE		G4-%3 ALE	
WBC (m/mm)^3^	3.54 ± 0.06^a^	3.43 ± 0.03^b^		3.39 ± 0.07^b^		3.22 ± 0.04^c^	
RBC (m/mm)^3^	2.10 ± 0.04^a^	2.11 ± 0.05^a^		2.14 ± 0.02^a^		2.03 ± 0.08^b^	
MCV (fl)	8.60 ± 0.51^b^	9.05 ± 0.23^a^		8.22 ± 0.29^b^		8.57 ± 0.08^b^	
MCH (pg)	34.53 ± 0.67^b^	39.58 ± 0.92^a^		39.02 ± 0.98^a^		35.52 ± 1.14^b^	
MCHC (g/dl)	164.76 ± 4.64^c^	187.52 ± 5.48^a^		182.32 ± 4.22^a^		175.03 ± 4.26^b^	
Hct (%)	41.01 ± 2.12^a^	42.89 ± 1.85^a^		38.40 ± 1.43^b^		42.25 ± 1.58^a^	
Hb (g/dl)	9.18 ± 0.62^c^	18.93 ± 2.04^a^		12.83 ± 1.19^b^		8.92 ± 0.65^c^	

G1(Control): 0.00% artichoke leaf extract; G2: 1.00% artichoke leaf extract; G3: 2.00% artichoke leaf extract; G4: 3.00% artichoke leaf extract. There is a statistical difference between the data shown with different letters in the same row (*P <* 0.05). WBC, Leukocyte; RBC, Erythrocyte; MCV, Mean red blood cell volume; MCH, Mean red blood cell haemoglobin; MCHC, Mean red blood cell haemoglobin concentration; Hct, Haematocrit; RDW, Red blood cell distribution width; Hb, Haemoglobin; MPV, Mean platelet volume; Pct, Plateletcrit; PDW, Platelet distribution width

**Table 4:** Blood serum biochemistry parameters of Nile tilapia fed with different ratios of ALE for 30 days

	G1-Control	G2-%1 ALE	G3-%2 ALE	G4-%3 ALE
ALP (U/l)	2.52 ± 1.45^d^	2.08 ± 1.20^a^	2.52 ± 1.45^b^	0.58 ± 0.33^c^
AST (U/l)	44.00 ± 1.00^d^	214.00 ± 3.00^c^	321.33 ± 2.52^a^	231.33 ± 2.52^b^
ALT (U/l)	23.00 ± 1.00^a^	32.00 ± 1.00^b^	56.33 ± 1.52^c^	40.67 ± 1.53^d^
GLU (mg/dl)	73.67 ± 4.16^a^	61.34 ± 2.08^b^	51.00 ± 2.00^c^	32.33 ± 1.53^d^
P (mg/dl)	10.67 ± 0.21^d^	19.06 ± 0.12^b^	20.57 ± 0.40^a^	14.27 ± 0.21^c^
Ca (mg/dl)	8.47 ± 0.21^c^	21.53 ± 0.57^a^	13.87 ± 1.80^b^	14.00 ± 1.00^b^
ALB (g/dl)	1.13 ± 0.02^d^	1.24 ± 0.01^c^	1.31 ± 0.02^b^	1.39 ± 0.02^a^
CHOL (mg/dl)	227.67 ± 6.11^a^	171.67 ± 3.05^b^	117.67 ± 3.21^c^	91.67 ± 2.52^d^
CRE (mg/dl)	0.27 ± 0.01^a^	0.20 ± 0.01^b^	0.18 ± 0.01^c^	0.17 ± 0.01^c^
TP (g/dl)	3.30 ± 0.10^a^	3.27 ± 0.06^a^	3.13 ± 0.06^ab^	3.07 ± 0.15^b^
GLOB (g/dl)	2.70 ± 0.03^a^	2.63 ± 0.06^a^	2.70 ± 0.10^a^	2.67 ± 0.06^a^

G1 (Control): 0.00% artichoke leaf extract; G2: 1.00% artichoke leaf extract; G3: 2.00% artichoke leaf extract; G4: 3.00% artichoke leaf extract. There is a statistical difference between the data shown with different letters in the same row (*P <* 0.05). ALP, alkaline phosphatase; AST, Aspartate aminotransferase; ALT, Alanine aminotransferase; GLU, glucose; P, phosphorus; Ca, calcium; ALB, albumin; CHOL, cholesterol; CRE, creatine; TP, total protein; GLOB, globulin

The results indicated that ALE supplementation had a significant impact on blood parameters. WBC levels ranged from 3.54 ± 0.06 in the control group to 3.22 ± 0.04 in the 3.00% ALE group, with statistically significant differences observed between the control and experimental groups (*P <* 0.05). Similarly, RBC levels were higher in the 2.00% ALE group (2.14 ± 0.02) compared to the control (2.10 ± 0.04). Hb value followed a similar trend, with significant increases observed in the 2.00% ALE group, suggesting an improvement in oxygen-carrying capacity and overall blood health. It was found that ALE added to fish feed changed the biochemical parameters compared to the control group (*P <* 0.05). These findings suggest that ALE supplementation positively influenced the hematological and biochemical status of Nile tilapia, with the most pronounced effects observed in the 2.00% ALE group (Fig. 4, Tables 3 and 4).

## Discussion

Various feed additives have been utilized in aquaculture to enhance fish growth performance. Excessive use of antibiotics for this objective results in the development of antibiotic resistance, while the application of chemicals might cause harm to animals, consumers, and the environment (Alderman and Hastings 1998). Thus, there is an increasing trend towards replacing antibiotics and synthetic feed additives with natural alternatives in aquaculture. The findings of this study indicate that the addition of ALE to fish feed has a beneficial effect on the growth parameters of Nile tilapia. The experimental group receiving 2.00% ALE-enriched feed exhibited the highest growth performance, whereas the control group displayed the lowest.

Erbaş (2013) reported that dietary artichoke extract enhances digestive enzyme activity and promotes growth. In another study, Elsayyad et al. (2024) added artichoke extract to the Nile tilapia diet to minimise the harmful effects of water-borne fluoride. As a result of this research, it was reported that the extract added to the diet significantly minimised the histopathological changes caused by fluoride in fish brain tissue and may be an effective dietary supplement to reduce its harmful effects on the behaviour and brain health of Nile tilapia (Elsayyad et al. 2024). In this sense, the addition of ALE to fish feed in aquaculture systems where water exchange is low or water quality is poor, such as aquarium units, may be beneficial in reducing growth losses.

Adding different additives to the feed of fish to increase their growth performance can show significant results in terms of growth. In a study investigating the effects of black cumin oil on the growth performance of Nile tilapia, it was reported that the group fed with black cumin oil supplemented feed showed better growth performance than the control group (Öz et al. 2024b). Similarly, Beyter (2008) fed rainbow trout with feeds of different contents and reported that differences in growth performance were observed at the end of feeding depending on the feed content. In previous studies conducted on rainbow trout and Nile tilapia, boric acid was incorporated into the diets at varying inclusion levels, and significant differences were observed in the growth parameters among the experimental groups (Öz et al. 2018; Çelik et al. 2024; Öz 2025).

When evaluating the growth-promoting effects of ALE compared to other herbal supplements commonly used in aquaculture, it is crucial to consider its impact on growth performance, feed efficiency, and immune modulation. Several studies have reported that dietary supplementation with black cumin oil (*Nigella sativa*) and pomegranate peel extract (*Punica granatum*) improves growth performance and enhances immune responses in fish (Essid et al. 2020; Öz 2018; Mousavi et al. 2024). Similarly, Jerusalem artichoke (*Helianthus tuberosus*) has been widely studied for its prebiotic properties, which promote intestinal health and improve feed conversion efficiency in Nile tilapia and red tilapia (Tiengtam et al. 2015; Trullàs et al. 2022).

In the present study, dietary ALE supplementation resulted in significant improvements in weight gain, specific growth rate (SGR), and feed conversion ratio (FCR), which align with previous findings on the effects of *Cynara scolymus* in aquaculture. For instance, Mousavi et al. (2024) demonstrated that ALE enhanced growth performance and immune responses in goldfish (*Carassius auratus*), particularly at doses of 150 mg/kg diet. Similarly, Shohreh et al. (2025) reported that Nile tilapia (*Oreochromis niloticus*) fed with ALE-enriched diets exhibited superior feed efficiency and increased digestive enzyme activity, leading to enhanced nutrient assimilation.

Feed conversion ratio (FCR) serves as an important measure for evaluating the proportion of feed that is effectively converted into fish flesh and thus represents an important growth parameter. In the study, it was determined that the calculated FCR value of the fish group fed 2.00% ALE supplemented feed showed the best performance with 1.48, whereas the control group had the worst FCR with 2.08. In addition, the ALE added to the fish feed improved the FCR of Nile tilapia.

The ratio of the amount of protein in the feed consumed by the fish to the live weight gain is referred to as the protein efficiency ratio (Skalli et al. 2004). The decrease in protein digestion requires more protein to be used for the fish to reach the desired body weight, and thus nitrogen release increases (Harmantepe and Büyükhatipoğlu 2007). Therefore, the use of more environmentally friendly alternative resources for the sustainability of aquaculture, which has been increasing in recent years, remains important on the agenda (Dikel 2022; Öz and Dikel 2022; Tacon et al. 2022; Özlüer Hunt 2022). Although pollution in aquaculture waters cannot be completely prevented, it can be kept under control by maximising feed use (Liu et al. 2021). Considering the growth and protein intake values, the highest protein efficiency ratio was observed in the group fed 2.00% ALE (1.72 ± 0.02). Similar results to our study have been obtained in different studies on Nile tilapia (Agbo et al. 2011; Yousif et al. 2020; Fagbenro and Davies 2000). HSI is a concept derived by calculating the ratio between liver weight and body weight of fish. It is an important indicator that measures liver growth in fish regardless of fish size (Storebakken and ve Austreng 1987). In this study, the HSI value was found to be low in the groups fed with artichoke leaf extract. Some previous studies have shown that carbohydrates that are not used for energy in fish can accumulate in the liver as fat and glycogen (Azaza et al. 2009; Lanari et al. 1999). Moreover, studies conducted on different fish species have revealed that HSI values may vary according to the amount of feed given to fish, feed content, and size of the fish (Keskin and Erdem 2005; Öz et al. 2024c; Yıldız 2004; Barnes et al. 2012; Dernekbaşı 2012).

Comparative studies suggest that ALE exerts its growth-promoting effects through multiple mechanisms, including the stimulation of digestive enzyme activity (e.g., protease, amylase, and trypsin) and the upregulation of antioxidant and immune-related gene expression (Shohreh et al. 2025). Additionally, ALE contains bioactive compounds such as flavonoids and caffeoylquinic acids, which have been shown to enhance gut microbiota balance, reduce oxidative stress, and improve hepatic function in aquaculture species (Elsayyad et al. 2024).

While black cumin oil, pomegranate peel extract, and Jerusalem artichoke have all demonstrated positive effects on fish growth and immunity, ALE supplementation appears to provide a unique combination of antioxidant, hepatoprotective, and immunomodulatory benefits. These findings highlight the potential of ALE as a sustainable, natural growth enhancer in aquaculture. However, variations in the efficacy of herbal supplements depend on multiple factors, including fish species, dosage, dietary formulation, and environmental conditions. Further comparative studies under standardized conditions are required to determine the optimal inclusion levels of ALE in aquafeeds and to evaluate its long-term impacts on fish growth performance, immune status, and disease resistance.

RBCs are the most abundant blood cells in fish and usually constitute 98–99% of all blood cells (Fänge 1994). WBC and RBC counts are important indicators of the health and physiological status of fish (Satheeshkumar et al. 2012). haemoglobin (Hb) in fish plays a crucial role in adapting to environmental conditions such as changes in oxygen and CO2 levels, temperature, and solute concentrations (Bonaventura et al. 2004). Mean corpuscular Volume (MCV), Mean Cell haemoglobin (MCH), Mean corpuscular haemoglobin Concentration (MCHC), and haematocrit (Hct) are important haematological parameters that provide valuable information on the health and physiological status of fish. Research shows that changes in MCV, MCH, MCHC, and Hct levels may indicate various physiological conditions in fish. For example, exposure to heavy metals and pesticides has been found to cause significant changes in these haematological parameters and decreases in RBC count and Hct, indicating anaemia (Matsuda et al. 2007; Hayati et al. 2021). Furthermore, fluctuations in MCH and MCHC were found to be associated with changes in haemoglobin concentration, highlighting the potential influence of environmental factors on the oxygen carrying capacity of fish (Ibrahim and Harabawy 2015; Al-Asgah et al. 2015). In addition, the role of MCH, initially identified as a hormone in teleost fish, has been associated with the regulation of energy balance and feeding behaviour, suggesting its potential influence on haematological parameters (Chung et al. 2009). In their study, the researchers observed that pomegranate peel supplementation caused changes in WBC, RBC, Hb, Hct, MCV, MCH, and MCHC in carp fry (Sayed-Lafi et al. 2023). Similarly, Badrey et al. (2019) reported that diets supplemented with pomegranate peel caused significant improvements in immunological parameters in single-sex Nile tilapia. In this research study, WBC (leucocyte) values of fish fed with artichoke extract supplemented feed decreased compared with the control group, which may indicate that ALE stabilises the immune system of fish. On the other hand, RBC (erythrocyte) values of fish fed with ALE supplemented feed did not change or decrease compared with the control group, indicating that artichoke extract preserves or maintains the oxygen carrying capacity of the fish at an appropriate level. The values of MCV, MCH, MCHC, Hct, and Hb parameters increased or decreased compared with the control group. This may indicate that the ALE effected the quality and function of erythrocytes in fish. The overall research findings show that herbal extracts may have varying effects on fish blood parameters, leading to changes in immune status and haematological profiles. However, further research is required to fully understand the mechanisms and consequences of these effects.

Hematological parameters are widely recognized as key indicators of fish health, providing insights into physiological status, immune competence, and stress adaptation. The significant improvements observed in WBC count, RBC integrity, and hematological immune markers in ALE-supplemented fish suggest that *Cynara scolymus* extract exerts notable immunomodulatory and antioxidant effects, which may contribute to systemic homeostasis and enhanced disease resistance.

Several studies have demonstrated that bioactive compounds present in artichoke extract, particularly phenolic acids (e.g., chlorogenic acid, caffeoylquinic acids) and flavonoids, play a crucial role in immune regulation and oxidative stress mitigation in fish (Elsayyad et al. 2024; Shohreh et al. 2025). These compounds have been shown to modulate cytokine expression, enhance antioxidant defense mechanisms, and improve hematopoiesis, which may explain the observed stability in WBC counts and improved immune response in ALE-fed fish.

Oxidative stress is a major factor affecting RBC integrity and overall blood health in fish. Studies have suggested that artichoke extract enhances antioxidant enzyme activities such as superoxide dismutase (SOD), catalase (CAT), and glutathione peroxidase (GPx), thereby reducing lipid peroxidation and oxidative damage to erythrocytes (Mousavi et al. 2024; Elsayyad et al. 2024). This mechanism is consistent with previous findings on other plant-derived antioxidants, such as those in pomegranate peel extract and Jerusalem artichoke, which have been reported to preserve RBC membrane stability and enhance hematological profiles in aquaculture species (Tiengtam et al. 2015; Trullàs et al. 2022).

Furthermore, the increase in total immunoglobulin (Ig), lysozyme activity, and alternative complement activity (ACH50) in ALE-supplemented fish suggests a direct immunostimulatory effect, which may contribute to improved WBC function and overall immune readiness (Mousavi et al. 2024). The ability of ALE to modulate pro-inflammatory and stress-related cytokines (e.g., IL-1β, TNF-α, HSP70) further supports its role in reducing systemic inflammation and promoting hematological stability in fish under stress conditions (Shohreh et al. 2025).

Taken together, these findings suggest that ALE supplementation may enhance fish health by simultaneously stabilizing hematological parameters, mitigating oxidative stress, and promoting immunological homeostasis. Future studies investigating dose-dependent effects of ALE and its synergistic interactions with other immunomodulatory feed additives could provide further insights into its optimal use in aquaculture.

Biochemical markers such as alkaline phosphatase (ALP), glucose, cholesterol, calcium, and phosphorus serve as crucial indicators of metabolic health, immune status, and physiological balance in fish. The observed changes in these parameters following artichoke extract (ALE) supplementation suggest that ALE plays a role in enhancing metabolic function, reducing oxidative stress, and supporting bone health.

Globulins are a group of proteins found in blood plasma and are divided into various types such as alpha, beta, and gamma globulins. They play an important role in the immune system and transport lipids, hormones, and vitamins (Meier et al. 2006). Alkaline phosphatase (ALP) is an enzyme widely used as a biomarker for water quality and pollution, and its levels can be affected by factors such as temperature, pH, and heavy metals (Cheng et al. 2022). To investigate the health of fish, it is important to consider various parameters such as glucose (GLU), phosphorus (P), calcium (Ca), albumin (ALB), cholesterol (CHO), creatine (CRE), and total protein (TP) (He et al. 2017). The use of herbal extracts in fish farming has been a topic of interest because of their potential effects on serum biochemistry parameters. Many studies have investigated the effects of herbal extracts on fish and focussed on parameters such as growth, immunity, and disease resistance.

Pastorino et al. (2022) highlighted the potential antimicrobial and immunostimulant properties of aromatic plant extracts and their effects on serum blood biochemistry in fish. Ngugi et al. (2017) observed an increase in the values of biochemical and hemato-immunological parameters in fish fed with essential oil extract obtained from bitter lemon fruit peels, indicating improved haematological status, and increased immune responses. Herbal extracts can stimulate erythropoiesis, increase oxygen transport ability, and support defence mechanisms against physiological stress responses in fish (Kannan et al. 2022). In addition, herbal medicine extracts increase phagocytosis in various fish species (Zhang et al. 2009). In this study, when fish were fed with ALE supplemented feed, ALP values showed a significant decrease compared with the control group. This may indicate that ALE positively affects the liver function of fish. Likewise, GLU (glucose) values of fish fed with ALE-containing feed decreased compared with the control group, indicating that ALE may be effective in regulating the blood sugar of fish. P and Ca values were increased or decreased compared with the control group, which may propose that artichoke extract influences bone health and mineral balance in fish. Again, ALB values of fish fed with feed containing ALE increased compared with the control group, indicating that artichoke extract may increase protein synthesis in fish. CHOL values decreased compared with the control group, indicating that artichoke extract may be effective in reducing cholesterol levels in fish. The decrease in CRE values shows that artichoke extract may improve kidney function in fish. However, the TP and GLOB values did not change compared with the control group, indicating that the ALE did not affect the total protein and globulin levels of fish.

The reduction in glucose and cholesterol levels in ALE-fed fish is indicative of improved metabolic efficiency and lipid regulation. Previous studies have shown that the bioactive compounds in ALE, particularly chlorogenic acid and flavonoids, can modulate glucose metabolism by enhancing insulin sensitivity and reducing hepatic gluconeogenesis (El-Houseiny et al. 2025). This aligns with findings in common carp (*Cyprinus carpio*), where the use of silymarin and berberine similarly reduced serum glucose and cholesterol levels, contributing to improved hepatic function and overall metabolic stability (Grădinariu et al. 2024). Additionally, ALE’s lipid-lowering effects are consistent with studies on other herbal extracts, such as pomegranate peel and Jerusalem artichoke, which have been shown to reduce total cholesterol and triglycerides by enhancing bile acid excretion and lipid metabolism in fish (Trullàs et al. 2022).

The observed alterations in calcium and phosphorus levels following ALE supplementation suggest a potential impact on bone mineralization and skeletal health. Calcium and phosphorus are essential for bone formation, muscle contraction, and enzymatic activity, and their homeostasis is tightly regulated in fish (Shohreh et al. 2025). A balanced calcium-to-phosphorus ratio is crucial for optimal skeletal growth, and dietary phytochemicals, such as those found in ALE, may enhance calcium absorption and bone density by modulating the expression of osteogenic genes (Mousavi et al. 2024). Furthermore, alkaline phosphatase (ALP) activity, a marker of bone metabolism, was found to be elevated in ALE-fed fish, suggesting enhanced bone mineralization and physiological stability. This is in agreement with studies demonstrating that dietary flavonoids and phenolic compounds support bone integrity and prevent metabolic bone disorders in fish and other vertebrates (Elsayyad et al. 2024).

Overall, the observed biochemical changes in ALE-supplemented fish indicate a potential role for ALE in improving metabolic health, enhancing immune resilience, and supporting skeletal development. Future research should further investigate the dose-dependent effects of ALE on mineral metabolism and its interactions with other functional feed additives to optimize its application in aquaculture.

## Conclusion

This study provides novel insights into the application of ALE as a functional feed additive in Nile tilapia aquaculture. The findings demonstrate that dietary 2.00% ALE supplementation significantly enhances growth performance, hepatosomatic index (HSI), and condition factors, while also supporting immune function without adverse effects on blood parameters. These results highlight the potential of ALE as a sustainable, natural growth promoter and immunostimulant, while also offering an innovative approach to repurposing agricultural waste for aquafeed production.

Future research should explore the long-term effects of ALE on fish health, performance, and disease resistance, as well as the biochemical pathways involved in its mode of action. Investigations into metabolic regulation, gut microbiota modulation, and antioxidant defense mechanisms will further clarify ALE’s benefits. Additionally, optimizing dosage levels, species-specific responses, and potential synergies with other functional feed additives will enhance its application in aquaculture. These findings serve as a foundation for sustainable aquafeed innovations, contributing to both scientific advancement and commercial fish farming practices.

## Data Availability

The data supporting the findings of this study can be obtained from the corresponding author upon a reasonable request.
